# Colorectal Cancer Screening for Average-Risk North Americans: An Economic Evaluation

**DOI:** 10.1371/journal.pmed.1000370

**Published:** 2010-11-23

**Authors:** Steven J. Heitman, Robert J. Hilsden, Flora Au, Scot Dowden, Braden J. Manns

**Affiliations:** 1 The Department of Medicine, University of Calgary, Alberta, Canada; 2 The Department of Community Health Sciences, University of Calgary, Alberta, Canada; 3 Alberta Health Services - Cancer Care, Alberta, Canada; 4 Libin Cardiovascular Institute, University of Calgary, Alberta, Canada; McGill University, Canada

## Abstract

**Background:**

Colorectal cancer (CRC) fulfills the World Health Organization criteria for mass screening, but screening uptake is low in most countries. CRC screening is resource intensive, and it is unclear if an optimal strategy exists. The objective of this study was to perform an economic evaluation of CRC screening in average risk North American individuals considering all relevant screening modalities and current CRC treatment costs.

**Methods and Findings:**

An incremental cost-utility analysis using a Markov model was performed comparing guaiac-based fecal occult blood test (FOBT) or fecal immunochemical test (FIT) annually, fecal DNA every 3 years, flexible sigmoidoscopy or computed tomographic colonography every 5 years, and colonoscopy every 10 years. All strategies were also compared to a no screening natural history arm. Given that different FIT assays and collection methods have been previously tested, three distinct FIT testing strategies were considered, on the basis of studies that have reported “low,” “mid,” and “high” test performance characteristics for detecting adenomas and CRC. Adenoma and CRC prevalence rates were based on a recent systematic review whereas screening adherence, test performance, and CRC treatment costs were based on publicly available data. The outcome measures included lifetime costs, number of cancers, cancer-related deaths, quality-adjusted life-years gained, and incremental cost-utility ratios. Sensitivity and scenario analyses were performed. Annual FIT, assuming mid-range testing characteristics, was more effective and less costly compared to all strategies (including no screening) except FIT-high. Among the lifetimes of 100,000 average-risk patients, the number of cancers could be reduced from 4,857 to 1,782 and the number of CRC deaths from 1,393 to 457, while saving CAN$68 per person. Although screening patients with FIT became more expensive than a strategy of no screening when the test performance of FIT was reduced, or the cost of managing CRC was lowered (e.g., for jurisdictions that do not fund expensive biologic chemotherapeutic regimens), CRC screening with FIT remained economically attractive.

**Conclusions:**

CRC screening with FIT reduces the risk of CRC and CRC-related deaths, and lowers health care costs in comparison to no screening and to other existing screening strategies. Health policy decision makers should consider prioritizing funding for CRC screening using FIT.

*Please see later in the article for the Editors' Summary*

## Abstract 2

An economic analysis of different screening methods for detection of colorectal cancers suggests that in US or Canadian settings, screening with fecal immunochemical testing results in lower health-care costs as compared with other screening approaches.

## Editors' Summary

**Background:**

Colorectal (bowel) cancer is the second leading cause of cancer deaths for both men and women in North America. Colorectal cancer screening is an important means for reducing morbidity and mortality and fulfils the World Health Organization criteria for mass screening. However, a variety of CRC screening approaches are available. Colonoscopy is viewed as the gold standard of colorectal cancer screening as it has a high sensitivity for identifying adenomas and cancer and polyps can be removed during the screening examination. However, colonoscopy is associated with a number of complications and there are also barriers to access. Another type of test, the guaiac fecal occult blood test, has been shown to reduce mortality from colorectal cancer but this test has low sensitivity for identifying colorectal neoplasia, particularly adenomas. Fecal immunochemical tests, which also detect blood in the stool, have improved test performance characteristics (high sensitivity and specificity) and the potential to improve participation rates compared to guaiac fecal occult blood test and flexible sigmoidoscopy. Fecal DNA (a stool test, based on the detection of DNA shed by cancerous tissue) is another screening option, as is computed tomographic colonography (“virtual” colonoscopy), that might rival colonoscopy in detecting advanced adenomas and colorectal cancer but is expensive and requires a full colonic preparation.

**Why Was This Study Done?:**

In the absence of firm comparative evidence to guide the selection of any one screening modality and given the varied test performance characteristics and the significant differences in costs and resources associated with each, a robust cost-effectiveness analysis might help health policy makers in deciding whether or not to offer screening and if so, in selecting the most appropriate and cost effective screening modality. In this study the researchers conducted a full economic evaluation of all relevant colorectal cancer screening modalities in North America.

**What Did the Researchers Do and Find?:**

The researchers used an incremental cost-utility analysis, a sophisticated modeling technique, and two hypothetical patient cohorts (individuals with an “average risk,” i.e., no family history of colorectal cancer, aged 50–64 and 65–75) to compare guaiac-based fecal occult blood test or fecal immunochemical test annually (the researchers considered three distinct fecal immunochemical testing strategies on the basis of assays and collection methods taken from studies that have reported “low,” “mid,” and “high” test performance characteristics), fecal DNA every three years, flexible sigmoidoscopy or computed tomographic colonography every 5 years, and colonoscopy every 10 years. The researchers also included a no screening natural history arm as a comparison to each screening approach. For the baseline data of their model, the researchers used adenoma and colorectal prevalence rates from a recent systematic review and based screening adherence, test performance, and colorectal treatment costs on available data. The researchers found that annual fecal immunochemical testing with mid-range testing characteristics, was more effective and less costly compared to all strategies (including no screening). Using this screening modality, among the lifetimes of 100,000 average-risk patients, the number of cancers could be reduced from 4,857 to 1,393 and the number of deaths from colorectal cancer from 1,782 to 457, while saving CAN$68 per person. Although in the sensitivity and scenario analysis, screening patients using fecal immunochemical testing became more expensive than a strategy of no screening when the test performance of fecal immunochemical testing was reduced, or the cost of managing colorectal cancers was lowered, the researchers found that screening for colorectal cancer with fecal immunochemical testing remained the most economically attractive screening option.

**What Do These Findings Mean?:**

This model-based economic analysis found that fecal immunochemical testing is more effective and less costly than all other colorectal screening strategies, including the most commonly-used stool-based screening test, guaiac-based fecal occult blood testing, and no screening. Furthermore, this study suggests that annual screening with fecal immunochemical testing (assuming mid-range test performance characteristics) reduces the risk of colorectal cancer and colorectal cancer–related deaths, and lowers health care costs in comparison to all other screening strategies and to no screening. Therefore, health policy makers should consider prioritizing funding for fecal immunochemical testing as the screening modality for colorectal cancer.

**Additional Information:**

Please access these Web sites via the online version of this summary at http://dx.doi.org/10.1371/journal.pmed.1000370.

- Cancer.org has information for patients on colorectal cancer
- The US Centers for Disease Control (CDC) list colorectal screening guidelines
- The CDC also provides patient information on colorectal cancer Screening

## Introduction

As the fourth most common cancer and second-leading cause of cancer death among men and women [1], colorectal cancer (CRC) is an important health issue. CRC fulfills the World Health Organization (WHO) criteria for mass screening [2], and existing clinical practice guidelines recommend that average risk individuals begin screening at age 50 [3]–[6]. A variety of CRC screening modalities are available, including stool-based tests and radiological and endoscopic examinations of the colon. Colonoscopy has high sensitivity for identifying adenomas and cancer and permits the removal of polyps during a screening examination [7]. However, the risk of complications (including bleeding, perforation, and death) and barriers to access including limited availability and high patient-borne costs [8] diminish its appeal. The guaiac-based fecal occult blood tests (FOBTs) have been shown in randomized controlled trials (RCTs) to reduce CRC mortality [9]–[11]. However, FOBT has low sensitivity for identifying colorectal neoplasia, in particular adenomas. The fecal immunochemical tests (FITs) have improved test performance characteristics [12] and potential to improve participation rates compared to FOBT and flexible sigmoidoscopy [13]. A third type of stool test, based on the detection of DNA shed by neoplastic tissue (fecal DNA) is also available [14],[15]. Lastly, computed tomographic colonography (CTC) or “virtual” colonoscopy is a promising new modality [6]. Although recent studies [16]–[18] have shown CTC to rival colonoscopy in detecting advanced adenomas and CRC, CTC is expensive, requires a full colonic preparation, and the available cost-effectiveness data have been contradictory [19]–[21].

In light of the rapidly rising costs of chemotherapy for CRC [22], and evidence that CRC mortality can be reduced by screening [9]–[11], population-based screening programs for average risk individuals are being considered in several countries. In the absence of firm comparative evidence to guide the selection of any one modality, the practice in some jurisdictions has been to recommend choice among the available screening options [3]–[5]. However, some countries do not support population-based CRC screening and many with organized programs do not offer choice [23]. Given the varied test performance characteristics and the significant differences in costs and resources associated with each, health care decision makers should consider the results of cost-effectiveness analyses when deciding whether or not to offer screening and in selecting the most appropriate screening modality.

There have been several previous economic analyses of CRC screening [24], though recent studies have failed to consider all potentially relevant strategies including CTC [25],[26] and FIT [27]. Furthermore, a wide range of FIT test performance has been reported, the impact of which requires further exploration in cost-effectiveness analyses. Finally, many studies have not considered current CRC treatment costs, nor the different nonmedical costs between CRC screening strategies, both of which may be important. Given these limitations, we performed a full economic evaluation of all relevant CRC screening modalities in North America, and present our results in a transparent fashion to assist medical decision makers.

## Methods

### Overview

An incremental cost-utility analysis was performed comparing the following CRC screening modalities: guaiac-based FOBT, FIT, fecal DNA, colonoscopy, flexible sigmoidoscopy, and CTC. These modalities were compared to each other and to a no screening natural history arm among average-risk individuals, aged 50 to 75 y. Two average-risk age-stratified patient cohorts were simultaneously modelled: people aged 50–64 and 65–75. In the base case, screening was assumed to continue from age 50–75, but the analysis continued over the lifetime of the cohorts. Average risk was defined as asymptomatic individuals with no personal or family history of CRC or adenomatous polyps and no history of preexisting medical conditions known to increase the risk of CRC (e.g., inflammatory bowel disease).

Although we acknowledge that many jurisdictions are already committed to CRC screening, we included a no screening strategy given that, despite widespread screening recommendations, the majority of individuals are not being screened [28]. In the base case analysis, costs were those relevant to a publicly funded health care system and included patient time and travel costs in keeping with recent guidelines [29]. Consistent with contemporary guidelines and the perspective of the publicly funded health care system, costs resulting from lost productivity were not considered [29]. Given the impact of CRC on both quantity and quality of life, health benefits were measured in quality-adjusted life-years (QALYs) gained over a lifetime horizon. Future costs and benefits were discounted at 5% annually [29]. Base case analyses were performed using Markov cohort simulation; second order probabilistic sensitivity analysis was used to derive 95% confidence intervals around mean costs and QALYs, and for probabilistic sensitivity analysis (see below). First order Monte Carlo simulation was used to estimate CRC incidence and death rates and the number of primary screening tests and colonoscopies required. Incremental analyses (expressed as the cost per QALY gained) were performed by rank ordering all competing strategies by increasing cost after eliminating strategies that were more costly and less effective (i.e., dominated).

### Model Validation

Consistent with guidelines for good modeling in health care [30], the validity of our model was formally established including extensive “debugging” exercises and calibration to published clinical datasets [9]–[11]. Gastroenterologists, including two of the authors (SJH and RJH), carefully reviewed the structure and flow of the model. The model was also reviewed by Alaa Rostom, Gastroenterologist and Medical Director at the Forzani and MacPhail Colon Cancer Screening Centre in Calgary, Alberta. Ultimately, it was determined that the model had good face validity. After ensuring that there were no syntactical errors, we first calibrated the model's no screening arm against the no screening control arms of the landmark FOBT RCTs [9]–[11]. For this we used baseline adenoma and CRC prevalence rates from a contemporary meta-analysis [31] and ensured that the number of cancers and cancer deaths generated by our model closely approximated the control arms of the clinical trials over an identical follow-up period. We next ensured that the number of cancers and cancer deaths predicted by the FOBT screening arms closely approximated those noted within the FOBT arms of the FOBT RCTs. All of the other strategies were validated in a similar fashion assuring face validity and calibration. Finally, we also compared our CRC and CRC death rate with those generated by another validated decision analytical model, noting near perfect correlation [32].

### Computer Simulation Model

**Figure 1:**
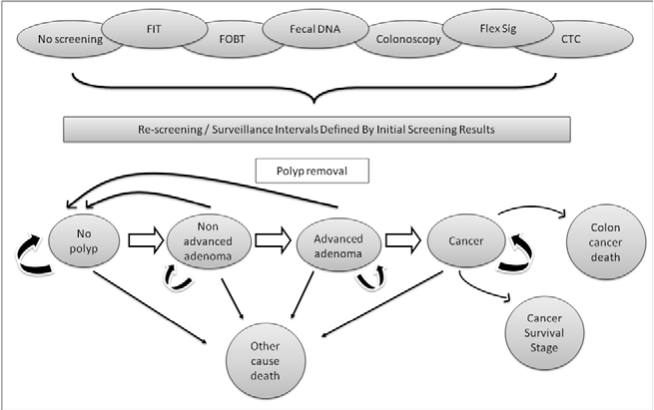
Model bubble diagram. This diagram depicts the general health states and flow through the model.

The Markov model was constructed using decision analysis software (TreeAge Pro Suite 2007). It was assumed that all CRCs arise through the following sequence: normal colon → nonadvanced adenoma → advanced adenoma → CRC. Nonadvanced adenomas were defined as tubular adenomas <10 mm in size. Advanced adenomas comprised any adenoma ≥10 mm regardless of histology, and adenomas <10 mm containing at least 25% villous component and/or high grade dysplasia. We considered several general health states, including (1) alive with no prevalent or prior history of adenomas or CRC, (2) alive with a missed adenoma, (3) alive with a missed asymptomatic CRC, (4) alive with a missed CRC after presenting with symptoms, (5) alive with a CRC found by screening, (6) alive post polypectomy, and (7) dead. Each year (1-y cycle length), individuals with or without adenomas or CRC could either remain in the same health state, progress to another health state, or die (Figure 1).

In the base case, screening was offered annually for FOBT and FIT, every 3 y for fecal DNA, every 5 y for flexible sigmoidoscopy and CTC, and every 10 y for colonoscopy. Once a patient was diagnosed with either an adenoma or CRC, the model's design permitted subsequent surveillance with colonoscopy at either 3- or 5-y intervals depending on the results of the last colonoscopy, consistent with current guidelines [4]–[6]. Screening and surveillance commenced at age 50 and stopped at age 75.

### Data Inputs

#### Risk of polyps and CRC and the adenoma-carcinoma sequence

We based our prevalence estimates of adenomatous polyps and CRC on a recent systematic review among those at average risk for CRC [31]. Age was determined to be an important source of heterogeneity in the pooled estimates [31], and thus the prevalence rates in our model were stratified into two age categories: 50–64 and 65–75 y (Table 1).

**Table 1:** Base case model inputs and ranges considered.

Variable	Values	Range	References
**Age-dependent variables**			
50- to 64-y-old individuals			
Prevalence of nonadvanced adenomas	0.171	(0.10–0.25)	[31]
Prevalence of advanced adenomas	0.038	(0.02–0.05)	[31]
Prevalence of CRC	0.001	(0.0005–0.002)	[31]
Annual death risk	0.005	—	[35]
65- to 75-y-old individuals			
Prevalence of nonadvanced adenomas	0.173	(0.10–0.25)	[31]
Prevalence of advanced adenomas	0.082	(0.05–0.10)	[31]
Prevalence of CRC	0.007	(0.002–0.01)	[31]
Annual death risk	0.018	—	[35]
**Age-independent variables**			
Probability of annual transition from:			
No polyp to nonadvanced adenoma – no history adenoma/CRC	0.02	(0.01–0.03)	[32] a
No polyp to nonadvanced adenoma – history adenoma/CRC	0.038	(0.03–0.05)	[32] a
Nonadvanced to advanced adenoma	0.019	(0.01–0.03)	[32] a
Advanced adenoma to CRC	0.048	(0.03–0.07)	[32] a
**CRC 5-y mortality rates**			
Stage I	0.068	—	[36]
Stage II	0.175	—	[36]
Stage III	0.405	—	[36]
Stage IV	0.919	—	[36]
**CRC stage distributions**			
In unscreened patients who develop CRC, the proportion with:			
Stage I	0.145	(0.12–0.25)	[9]–[11]
Stage II	0.356	(0.34–0.39)	[9]–[11]
Stage III	0.280	(0.23–0.32)	[9]–[11]
Stage IV	0.219	(0.18–0.25)	[9]–[11]
In patients who have CRC found using FIT, FOBT, and FDNA, the proportion with:			
Stage I	0.305	(0.29–0.33)	[9]–[11]
Stage II	0.318	(0.30–0.35)	[9]–[11]
Stage III	0.243	(0.20–0.26)	[9]–[11]
Stage IV	0.134	(0.10–0.15)	[9]–[11]
In patients who have CRC found using colonoscopy, CTC, and flex sig, the proportion with:			
Stage I	0.425	(0.41–0.50)	[38],[46],[68]
Stage II	0.226	(0.22–0.26)	[38],[46],[68]
Stage III	0.267	(0.20–0.27)	[38],[46],[68]
Stage IV	0.082	(0.0–0.09)	[38],[46],[68]
**Screening adherence rates (all strategies)**			
1st screen	0.68	(0.30–0.80)	[9]–[11]
Subsequent screens	0.63	(0.10–0.80)	[9]–[11]
Probability of colonoscopy after positive CTC, FOBT, FIT, FDNA, or flex sig	0.81	(0.60–0.90)	[11]
**Risk of bleeding**			
Colonoscopy, diagnostic	0.0003	(0.0–0.009)	[69],[70]
Colonoscopy, therapeutic	0.005	(0.003–0.015)	[69]–[72]
**Risk of perforation**			
Colonoscopy, diagnostic	0.0009	(0.0005–0.002)	[70],[73]
Colonoscopy, therapeutic	0.0024	(0.001–0.005)	[70],[73]
Flexible sigmoidoscopy	0.0002	(0.0001–0.0004)	[32]
Risk of death after endoscopic perforation	0.049	(0.01–0.15)	[74]
**Patient utility**			
No CRC	0.91	—	[63]
Early CRC	0.74	—	[63]
Advanced CRC	0.46	—	[63]
**Discount rate**	0.05	—	[29]

a Minor adjustments were applied to the rates used in the US Multi-Society Task Force model [32] such that the total of our baseline prevalence of CRC plus the number of new CRCs developing in our natural history arm closely approximated the number of CRCs observed in the control arms of the FOBT RCTs [9]–[11].

Not all polyps are adenomatous. However, determining a polyp's histology generally requires that it be biopsied or removed. As a result, some polypectomies expose patients to complications without reducing the risk of CRC. We estimated that 41% [33] of polyps <10 mm were adenomatous compared to 82% of polyps ≥10 mm (Table 1) [16]. Screening guidelines recommend that all polyps be removed at the time of a colonoscopy to determine histology and establish an appropriate surveillance interval. Although some advocate for ignoring polyps <5 mm in size found on CTC, we assumed that all patients with polyps found on CTC regardless of size would be referred for colonoscopy. The risk of proximal adenomatous polyps and CRC is increased among those with adenomatous polyps in the left colon [34]. As such, we assumed that patients with left-sided adenomas found on flexible sigmoidoscopy would be referred for colonoscopy consistent with general clinical practice.

The rate of progression of adenomatous polyps is not well established. We initially chose progression rates that were consistent with other published models [32], and made small adjustments to these rates to ensure that the total number of CRCs in our natural history/no screening strategy closely approximated the number of CRCs found in the control arms of the FOBT trials [27].

#### Mortality

Death occurred according to either age-dependent population mortality rates observed for Canadians [35] or based on the mortality rates observed for patients with CRC according to their stage at diagnosis (Table 1) [36]. Those with CRC found through screening were assumed to have improved survival over patients presenting with symptomatic cancer, on the basis of a more favorable stage distribution (i.e., more early stage cancers) at diagnosis (Table 1).

#### Screening adherence

Adherence is important to the overall effectiveness of a screening program. Even in a randomized trial comparing annual FOBT with no screening, only 68% of patients who were randomized to FOBT actually completed the initial screen and 63% were compliant with subsequent rescreening. Moreover, for patients with positive FOBT results, only 81% had a colonoscopy [11]. We adopted these imperfect adherence rates and assumed in the base case that adherence would be the same across strategies (Table 1).

#### Test performance characteristics of the CRC screening strategies

The only method for properly assessing the test performance of a given screening modality is to compare it with a reference standard in all cases. Although colonoscopy is not infallible [7], it remains the accepted gold standard for evaluating the entire colon. Therefore, the base case sensitivities and specificities for polyps and CRC for each of the screening modalities were taken from the literature following a thorough search for properly designed studies that included at least a full colonoscopy in all individuals (Table 2). For the stool-based tests and for CTC, the test performance characteristics were considered on a per person basis.

**Table 2:** Base case test performance characteristics for the screening modalities.

Screening Modality	Sensitivity			Specificity
	Nonadvanced Adenoma	Advanced Adenoma	Cancer	
FOBT-low [38]	0.052	0.107	0.129	0.952
FOBT-high [14]	0.030	0.074	0.500	0.980
FIT-low [39],[40]	0.07	0.224	0.660	0.950
FIT-mid [41]	0.180	0.540	0.810	0.960
FIT-high [42]	0.180	0.610	0.940	0.910
Colonoscopy [18],[49],[50],[75]	0.850	0.875	0.966	1.000
Colonoscopy after positive CTC	0.900	0.970	0.99	1.000
CTC [16]	0.760	0.900	0.966	0.890
Flexible sigmoidoscopy [44],[45],[46]	0.650	0.750	0.750	1.000
FDNA-SDT2 [14]	0.040	0.447	0.580	0.840
FDNA-SDT1 [38]	0.076	0.151	0.516	0.944

#### Stool-based tests

Given significant differences even between the alternative stool-based screening tests themselves (often due to different collection methods or assay types), it would not be appropriate to consider them as a class [37]. As such, we modeled different test performance scenarios for each test. A low [38] and high [14] performance level was modeled for FOBT tests that have reported in the literature (FOBT-low and FOBT-high, respectively) and a low [39],[40], mid [41], and high [42] performance level was modeled for FIT assays that have been reported in the literature (FIT-low, FIT-mid, and FIT-high, respectively). The intent of modeling different levels of test performance for FOBT and FIT was to represent the range reported in the literature. This range is greatest for FIT, likely due to differences in collection methods and assays (Table 2). FIT-low represents that reported by Morikawa et al. [39],[40] who studied the Magstream system with 1 d of stool collection. FIT-mid represents that reported by Nakama et al. [41] who used a 2-d method with the Monohaem system. FIT-high represents that reported by Levi et al. [42] who used the FlexSure OBT technology following 3 d of fecal collection. Both the first- [38] and second- [14] generation fecal DNA assays were modeled (FDNA-SDT1 and FDNA-SDT2, respectively).

#### Flexible sigmoidoscopy

Flexible sigmoidoscopy can evaluate the left colon to the splenic flexure, although this is not always possible [43]. Routine clinical practice is generally to perform a full colonoscopy in individuals found to have an adenomatous polyp on flexible sigmoidoscopy. As such, the sensitivity of flexible sigmoidoscopy includes the additional lesions found by colonoscopy in patients identified as having an adenoma on flexible sigmoidoscopy [44]–[46].

#### CTC and colonoscopy

Landmark studies [16],[18] that employed segmental unblinding methodology [47] provided the base case test performance estimates for both CTC and colonoscopy when possible. The sensitivity and specificity of CTC for polyps ≥10 mm was taken from the National CT Colonography Trial of the American College of Radiology Imaging Network (ACRIN) [16], a large multicenter study of CTC among primarily average-risk individuals. Polyps <5 mm were not reported in this study or other large cohorts of average risk individuals. However, we optimistically assumed that the sensitivity reported for 6–9 mm polyps would be the same for all polyps <10 mm. In a sensitivity analysis we reduced the sensitivity of polyps <10 mm to that reported in a meta-analysis of CTC that included higher risk patients [48]. The sensitivity of colonoscopy for polyps ≥10 mm was taken from the study of Pickhardt et al. [18], which reported the test performance of both CTC and colonoscopy based on segmental unblinding. As this study also did not report data for polyps <5 mm, the sensitivity of colonoscopy for polyps <10 mm was taken from two back-to-back colonoscopy studies (Table 2) [49],[50].

#### Screening-related risks

Flexible sigmoidoscopy and colonoscopy are associated with risks including bleeding, perforation, and rarely, death (Table 1). Even though CTC is less invasive than colonoscopy, colonic perforations have been reported [51]–[53], though many of the small CTC induced perforations diagnosed with the CT in asymptomatic individuals may not be clinically important. We assumed a low risk of CTC-induced perforation in the base case analysis [51],[52], and that this would never result in death (Table 1).

#### Costs

***Costs related to screening.*** All costs are reported in 2008 CAN$. The direct costs of flexible sigmoidoscopy and colonoscopy, as well as costs attributed to bleeding and perforation complications [54], were based on local estimates derived from the Calgary Health Region costing database [55] and included the nonphysician costs (capital, nursing, drugs, and cleaning costs) and the physician fees for the procedure (Table 3). CTC for primary CRC screening is not currently part of the schedule of medical benefits in any province in Canada. The direct costs of CTC were therefore conservatively assumed to be the same as that of a CT abdomen/pelvis, likely an underestimate (Table 3). We assumed that stool-based screening would be offered at a person's annual visit to their general practitioner, and as such, we only considered the cost of the screening kit and related laboratory/processing costs (Table 3).

**Table 3:** Base case direct health care costs and nonmedical costs and ranges considered.

Variable	Values CAN$	Range CAN$	References
FOBT^a^	12	6–18	[27]
FIT	19	10–30	[76]
Colonoscopy, diagnostic^b^	857	500–1,200	[27]
Colonoscopy, therapeutic^c^	999	700–1,700	[27]
CTC	582	440–730	[27]
FDNA	336	200–500	[25]
Flex sig	650	400–900	Determined locally
Bleeding complication	3,194	(2,400–4,000)	[54]
Perforation complication	31,223	(23,500–39,000)	[54]
Total cost of managing CRC			Determined locally and [58]–[62]
Stage I CRC	25,049	**—**	
Stage II CRC	36,143	**—**	
Stage III CRC	96,768	**—**	
Stage IV CRC	134,014	**—**	
Nonmedical^d^			[8],[56],[57],[77]
FOBT	36	(25–50)	
FIT	36	(25–50)	
FDNA	36	(25–50)	
Colonoscopy	308	(200–450)	
CTC	105	(100–200)	
Flex sig	105	(100–200)	

a FOBT: includes cost of FOBT kit (CAN$5), processing (CAN$7).

b Diagnostic colonoscopy: includes physician cost of diagnostic colonoscopy (CAN$327), and nonphysician cost of colonoscopy (CAN$530).

c Therapeutic colonoscopy: includes physician cost of therapeutic colonoscopy (CAN$401), and nonphysician cost of therapeutic colonoscopy (CAN$598).

d Includes patient ± caregiver time and travel costs, but excludes productivity losses [29].

For all screening modalities, we included the relevant patient ± caregiver time and travel costs (nonmedical costs), on the basis of available surveys for flexible sigmoidoscopy, colonoscopy, FOBT, and CTC (Table 3) [8],[56],[57]. The nonmedical costs of FIT and fecal DNA were assumed to be the same as FOBT. In the base case, we did not consider the capital costs of initiating or administering a screening program and thus assumed that screening would be opportunistic in all strategies.

***Costs related to managing CRC.*** Existing published data on the total costs of managing patients with CRC are outdated. We assumed that the cost of surgery for CRC has remained relatively stable and thus based our surgical costs on a Canadian study reporting 1998 figures, inflation adjusted to 2008 dollars [58].

In contrast, the cost of treating CRC with chemotherapy has increased substantially because of the development of more expensive agents [22]. To estimate the cost of chemotherapy provided for advanced CRC, we used data from the Canadian Inter-Provincial Joint Oncology Drug Review (JODR) Process [59]. These estimates were the average stage-based treatment costs for chemotherapy, taking into account that not all patients would be eligible for or would comply with treatment. Patients with stage IIB disease (∼50% of stage II patients) are generally managed with adjuvant chemotherapy using eight cycles of capecitabine [60],[61]. First line therapy for patients with stage III CRC was assumed to be 6 mo of oxaliplatin-based therapy [62]. Considering the most recent clinical trials and assumed standards of care, the average patient with stage IV CRC received approximately 10 mo of infusional fluorouracil (5-FU), leucovorin, and oxaliplatin (FOLFOX) in combination with bevacizumab, followed by 14 doses of infusional 5-FU, leucovorin, and irinotecan (FOLFIRI). Those lacking *K-Ras* mutations were assumed to go on to receive 4 mo of anti-epidermal growth factor receptor–based inhibition therapy. We did not include the potential costs of liver metastectomy among stage IV patients, or the cost of preoperative radiation therapy in patients with operable rectal cancer.

#### Valuing health benefits

Health benefits were measured in terms of QALYs gained. We obtained utilities for relevant health states on the basis of a study that used a standard gamble exercise in patients with a previous history of CRC or polyps who were presented with stage-dependent outcome states for CRC (Table 1) [63].

### Sensitivity Analysis

Allowance for uncertainty in the base case polyp and CRC prevalence estimates, mortality assumptions, screening test performance characteristics, screening-related risks, and costs were considered through the use of univariate and probabilistic sensitivity analyses. A number of scenario analyses were also included. We considered a scenario in which the additional costs of biologic chemotherapies for advanced stage CRC were excluded. We also examined scenarios where FIT was offered every 2 y instead of annually and analyzed our results without nonmedical costs. We assessed the impact of differential adherence rates across strategies at the initial screening encounter. For this analysis, we used the adherence rates determined by Hol et al. in a RCT comparing participation rates of FOBT, FIT, and flexible sigmoidoscopy in a screening population [13]. As Hol et al. [13] did not study fecal DNA, colonoscopy, or CTC, we assumed that fecal DNA would have the same adherence as FIT due to its comparable simplicity for patients, and we assumed that colonoscopy would have the same adherence as flexible sigmoidoscopy. We also assessed the impact of lower subsequent adherence for the annual stool-based tests, since screening noncompliance may be more prevalent with an annual test compared to one offered less frequently. To do this assessment, we examined scenarios with decreased FOBT and FIT follow-up adherence.

Because we did not include any administrative costs for any of the CRC screening programs, we performed a sensitivity analysis to assess the impact of including administrative costs for the various screening tests. We were unable to identify a document that has reported the setup and operating costs for a population-based CRC screening program, but it is possible that programs that screen annually (i.e., stool-based tests) might have higher administrative costs than ones that screen patients every 10 y (i.e., colonoscopy). We provide sensitivity analyses varying the administrative costs per screening test between CAN$10 and CAN$50 to determine the impact on the results, making the assumption that programs screening more frequently will incur higher administrative costs.

To address limitations in classic univariate sensitivity analysis, we also performed probabilistic sensitivity analysis, which allows for the simultaneous sensitivity analysis of all variables over their plausible range [64],[65]. It does so by replacing estimates of probabilities, utilities, and costs with specific probability distributions, which are based on the reported means and variances for each variable. Statistical distributions were created around all of the variables for which there was substantial measurement uncertainty, including use of a beta distribution for proportions (i.e., mortality, proportion of patients with Stage I, II, III, and IV cancer), use of a normal distribution for normally distributed variables (i.e., certain costs and utility measures), log-normal distribution for skewed variables (i.e., certain costs), and triangular distributions for variables with a range, but no statistical distribution (i.e., adenoma transition over time, probability of adherence). Given that sensitivity and specificity are linked variables that do not vary independently (linked via receiver operating curves that were unavailable), these variables were not included within the probabilistic analyses—as noted above, the sensitivity and specificity of the various screening tests were subjected to wide sensitivity analysis using the testing characteristics provided by different primary studies.

## Results

### Base Case Analysis

Annual CRC screening using FIT, assuming mid-range test performance characteristics, was the preferred strategy for average risk individuals in the base case analysis (Table 4). It was more effective and less costly than almost all of the other strategies including no screening. Only FIT when assuming even better test performance characteristics (i.e., FIT-high) produced more QALYs and resulted in fewer CRCs than FIT-mid, but at an additional cost of CAN$85,150 per QALY gained.

**Table 4:** Base case incremental cost per QALY gained for average risk patients (reported value compares strategy reported in the column with the strategy reported in the row).

Screening	Average Costs (CAN$) (95% CI)^a^	Average QALYs (95% CI)^a^	Incremental Cost Per QALY Gained										
			FIT-Mid	No Screening	FIT-High (CAN$)	FIT-Low (CAN$)	FOBT-High (CAN$)	Colonoscopy (CAN$)	FOBT-Low (CAN$)	Flex Sig (CAN$)	CTC (CAN$)	FDNA-SDT 2 (CAN$)	FDNA-SDT1 (CAN$)
FIT-mid	1,833 (1,275–1,924)	11.300 (11.29–11.30)	—	Dominated^b^	85,150	Dominated^b^	Dominated^b^	Dominated^b^	Dominated^b^	Dominated^b^	Dominated^b^	Dominated^b^	Dominated^b^
No screening	1,901 (1,641–2,226)	11.255 (11.24–11.26)	—	—	2,219	3,883	15,991	4,870	18,595	10,008	12,500	25,974	82,747
FIT-high	2,004 (1,353–2,207)	11.302 (11.29–11.31)	—	—	—	Dominated^b^	Dominated^b^	Dominated^b^	Dominated^b^	Dominated^b^	Dominated^b^	Dominated^b^	Dominated^b^
FIT-low	2,005 (1,519–2,020)	11.282 (11.27–11.29)	—	—	—	—	Dominated^b^	6,706	Dominated^b^	27,158	28,871	Dominated^b^	Dominated^b^
FOBT-high	2,084 (1,820–2,301)	11.267 (11.25–11.27)	—	—	—	—	—	573	25,341	7,247	11,137	36,044	Dominated^b^
Colonoscopy	2,100 (1,536–2,120)	11.296 (11.29–11.30)	—	—	—	—	—	—	Dominated^b^	Dominated^b^	Dominated^b^	Dominated^b^	Dominated^b^
FOBT-low	2,195 (1,892–3,375)	11.271 (11.26–11.28)	—	—	—	—	—	—	—	3,325	8,617	42,870	Dominated^b^
Flex sig	2,263 (2,136–2,433)	11.291 (11.28–11.30)	—	—	—	—	—	—	—	—	32,489	200	Dominated^b^
CTC	2,409 (2,124–2,508)	11.296 (11.27–11.28)	—	—	—	—	—	—	—	—	—	Dominated^b^	Dominated^b^
FDNA-SDT2	2,491 (2,187–2,644)	11.278 (11.27–11.28)	—	—	—	—	—	—	—	—	—	—	Dominated^b^
FDNA-SDT1	2,720 (2,422–2,937)	11.265 (11.25–11.27)	—	—	—	—	—	—	—	—	—	—	—

a 95% confidence intervals (CIs) based on probabilistic sensitivity analysis using baseline statistical distributions around all uncertain variables.

b Dominated is defined as more costly and fewer QALYs compared with the strategy reported in the row.

Using base case estimates, over the lifetimes of a 100,000 patient cohort, 4,857 and 1,782 individuals would develop and die from CRC, respectively, if CRC screening was not undertaken (Table 5). This “no screening” strategy would be expected to cost an average of CAN$1,901 per patient. Annual screening with FIT-mid would reduce the overall number of cancers by 71% and CRC mortality by 74% while saving CAN$68 per patient. Compared with the most effective FOBT strategy, FIT-mid would be expected to reduce the number of cancers by 60%, and CRC mortality by 63%, while saving CAN$362 per person.

**Table 5:** Cancer outcomes and number of screening tests required during the lifetimes for a hypothetical 100,000 average risk patient cohort.

Screening Test	*n* Cancers Overall^a^	*n* Cancer Deaths	*n* Primary Screening Tests	*n* Colonoscopies	Cost Of Screening And Managing CRC (CAN$)
FIT-high	1,290	432	819,178	56,541	2,004
FIT-mid	1,393	457	822,077	53,909	1,833
CTC	1,796	593	188,315	58,354	2,409
Colonoscopy	1,825	624	155,210	N/A	2,100
Flex Sig	2,036	699	189,135	49,484	2,263
FIT-low	2,634	918	871,986	31,597	2,005
FDNA-SDT2	3,129	1,143	331,090	20,805	2,491
FOBT-low	3,457	1,250	889,168	21,805	2,195
FOBT-high	3,890	1,368	902,299	15,089	2,084
FDNA-SDT1	4,131	1,530	331,699	14,548	2,720
No screening	4,857	1,782	n/a	n/a	1,901

a *n* cancers overall include symptomatic and screen found CRC.

### Sensitivity Analysis

Under no circumstances did flexible sigmoidoscopy, FOBT, CTC, or fecal DNA appear attractive in comparison to other CRC screening modalities. As such, these strategies are not reported in our sensitivity analysis table (Table 6). Lowering the cost of CRC treatment by excluding the use of biologic chemotherapies resulted in a scenario where FIT-mid resulted in additional costs compared to no screening (CAN$163 per patient or CAN$3,691 per QALY gained). Increasing the cost of FIT testing by 50% had a similar effect; FIT-mid cost an additional CAN$105 per patient and was associated with a cost per QALY of CAN$2,375 compared to no screening. Biennial screening using FIT-mid increased the cost savings when compared to no screening. However, performing FIT less frequently also made it less effective.

**Table 6:** Sensitivity analysis.

Screening	Cost of Screening and Management (CAN$)^a^	QALY	Incremental Cost per QALY Gained (CAN$)^a,^ b
**Base case**			
FIT-mid	1,833	11.300	
No screening	1,901	11.255	(Dominated)^c^
FIT-high	2,004	11.302	84,876
Colonoscopy	2,100	11.296	(Dominated)^c^
**Lower stage III and IV cancer costs, including chemotherapy, but without biologics (Stage II CAN$35844, Stage III CAN$80,345, and stage IV CAN$99,574)**			
No screening	1,582	11.255	
FIT-mid	1,745	11.300	3,691
FIT-high	1,842	11.302	89,921
Colonoscopy	1,990	11.296	(Dominated)^c^
**Increase FIT direct cost by 50%**			
No screening	1,901	11.255	
FIT-mid	2,006	11.300	2,375
Colonoscopy	2,100	11.296	(Dominated)^c^
FIT-high	2,177	11.302	84,750
**Biennial FIT screening (versus annual FIT screening modeled in baseline analyses)**			
FIT-mid	1,736	11.289	
FIT-high	1,784	11.291	19,606
No screening	1,901	11.255	(Dominated)^c^
Colonoscopy	2,100	11.296	64,741
**Initial adherence 60% for FIT and fecal DNA, 50% for FOBT, 40% for CT colonoscopy, and 30% for colonoscopy ** [**13**]			
FIT-mid	1,815	11.299	
No screening	1,901	11.255	(Dominated)^c^
FIT-high	1,986	11.301	85,927
Colonoscopy	2,055	11.279	(Dominated)^c^
**Decrease subsequent adherence rates for FITs and FOBTs from 63% to 40%**			
FIT-mid	1,751	11.293	
FIT-high	1,839	11.295	38,536
No screening	1,901	11.255	(Dominated)^c^
Colonoscopy	2,100	11.296	300,609
**Decrease subsequent adherence rates for FITs and FOBTs from 63% to 20%**			
FIT-mid	1,752	11.283	
FIT-high	1,772	11.286	8,709
No screening	1,901	11.255	(Dominated)^c^
Colonoscopy	2,100	11.296	32,912
**CAN$10 administrative cost added for all screening tests**			
No screening	1,901	11.255	
FIT-mid	1,902	11.300	17
FIT-high	2,075	11.302	85,831
Colonoscopy	2,109	11.296	(Dominated)^c^
**CAN$50 administrative cost added for all screening tests**			
No screening	1,901	11.255	
Colonoscopy	2,143	11.296	5,903
FIT-mid	2,176	11.300	10,202
FIT-high	2,357	11.302	89,651

^a^Numbers rounded to nearest CAN$1.

b Each incremental value compares the value of that strategy to next most costly, nondominated, strategy.

c Dominated is defined as more costly and fewer QALYs compared with a comparator strategy.

When the initial adherence rates for each of the strategies was no longer assumed to be identical, FIT-mid remained dominant over no screening (Table 6). Assuming the base case initial adherence rates, when we dropped the adherence rates for subsequent screens for all of the annual fecal-based strategies, FIT-mid remained dominant over no screening. However, when subsequent adherence for FIT was dropped from 63% to 40%, both FIT-mid and FIT-high became dominant over no screening, and colonoscopy became the most effective strategy at a cost per QALY gained of CAN$300,609 compared to FIT-high. When subsequent adherence for FIT was decreased to only 20%, colonoscopy remained the most effective strategy, at a cost per QALY gained of CAN$32,912 compared to FIT-high (Table 6).

Finally, we performed a sensitivity analysis to assess the impact of higher administrative costs that might be associated with an annual screening program (i.e., FIT) compared to one offered less frequently (i.e., colonoscopy). We noted that FIT remained dominant over no screening unless the administrative costs were ∼CAN$10 per test. If administrative costs were CAN$30 per test, annual FIT was associated with a cost per QALY of CAN$3,120 compared with no screening. However, if the administrative costs were CAN$50 per test, then colonoscopy would be the preferred screening modality compared with FIT, and would be associated with a cost per QALY gained of CAN$5,903 compared with no screening.

**Figure 2:**
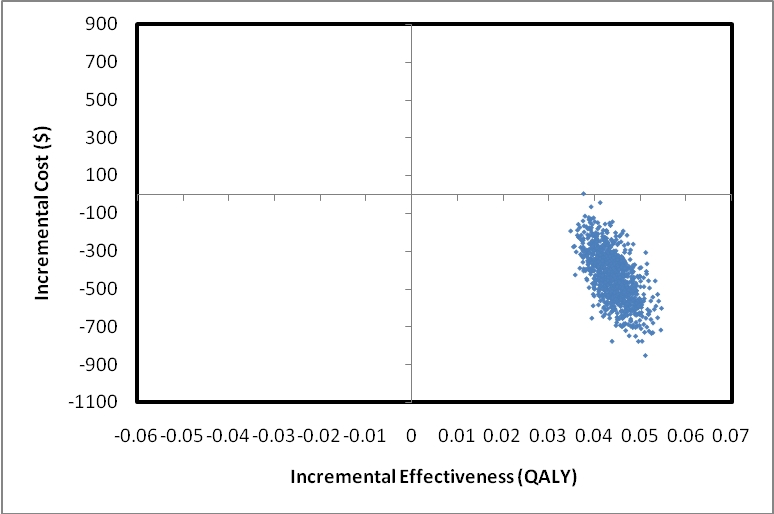
Probabilistic sensitivity analysis. An incremental cost-effectiveness scatterplot comparing FIT-mid with no screening in which the uncertainty in all model inputs has been tested simultaneously. Data points in the lower right quadrant reflect situations where FIT-mid is more effective and less costly than no screening.

Our probabilistic sensitivity analysis revealed that FIT-mid was cost saving and more effective compared with no screening in nearly 100% of the simulations performed, confirming the robustness of the results (Figure 2).

## Discussion

Our study demonstrates that annual screening with FIT, assuming mid-range test performance characteristics, is more effective and less costly than other CRC screening strategies, including the most commonly used stool-based CRC screening test, FOBT, and no screening. Among a cohort of 100,000 average risk individuals followed until death, 4,857 cancers and 1,782 cancer-related deaths would be expected with no screening. An annual FIT with high sensitivity for cancer (81%) and moderate sensitivity for advanced adenomas (54%) [41] could reduce costs and decrease the number of CRCs and cancer-related deaths to 1,393 and 457, respectively. Screening with FIT was also more effective at reducing cancer and cancer-related deaths at lower costs compared with FOBT.

FIT represents a significant advance over the traditional guaiac-based FOBTs, in large part due to FITs improved sensitivity for identifying adenomatous polyps. Our findings underscore the importance of identifying patients with advanced adenomas and preventing cancer through the identification and removal of precancerous polyps. Indeed, changing the sensitivity of FIT for cancer had relatively little impact on our results, whereas reducing the sensitivity of FIT for advanced adenomas from 54% to below 45% resulted in FIT no longer being cost saving compared with no screening.

Although it may seem counter-intuitive that screening with FIT could be even more effective than colonoscopy, this is due to the more frequent screening interval with FIT. In base case analyses, and consistent with current guidelines [3],[6], screening with FIT was done annually compared to every 10 y with colonoscopy. Therefore, even though the test performance of a single FIT test was inferior to colonoscopy, there were more opportunities to identify previously missed pathology with FIT compared to colonoscopy.

Our results are robust. FIT with mid-range performance (FIT-mid) remained optimal compared with no screening and all the other strategies except FIT with even better test performance (FIT-high) unless the cost of CRC treatment was reduced, or the sensitivity for advanced adenomas was decreased significantly. However, even with lower CRC treatment costs, FIT remained economically attractive. Many health jurisdictions now fund biologic chemotherapies for advanced-stage CRC and with further advances in CRC chemotherapy, it is unlikely that management costs for CRC will decrease [22]. In addition, our modeled CRC treatment costs were lower than those used in a recent US study that had similar results [25], lending further support to the notion that CRC screening can indeed save money.

It is possible that the administrative costs of annual screening programs such as FIT would be more expensive over the long-run compared with those offered every 5 or 10 y. As these data are not known, we did not consider administrative costs or the costs to build and staff additional screening centers in our primary analysis. However, in sensitivity analysis, we noted that FIT-mid remained cost saving if the administration costs were <CAN$10 per test, and remained attractive compared with colonoscopy even if the administrative costs per test were CAN$30 per test. It should also be noted that the additional infrastructure required to implement primary screening with CTC, flexible sigmoidoscopy, or colonoscopy would likely counterbalance a substantial portion of these additional administrative costs of an annual screening program.

We assumed in the base case that adherence would be identical across all of the CRC screening strategies. Although this may not be true, we are unaware of a study that has evaluated screening uptake for all of the strategies we considered. However, fecal-based screening does not require a bowel preparation, is associated with lower patient-borne costs, and is safe to perform, which may be more appealing to the general population. Furthermore, FIT does not require any dietary restrictions. Indeed, in a recent randomized trial, FIT was associated with higher screening uptake than flexible sigmoidoscopy and FOBT [13]. Of course, this finding only strengthens our conclusions as illustrated in our scenario analysis in which FIT had relatively higher adherence than all of the other strategies (Table 6). Recent data suggest that screening adherence with FOBT may drop by 50% after only 2 y in a biennial screening program [66]; this may affect programs with frequent screening (i.e., annual fecal-based strategies) to a greater extent than programs requiring less frequent screening (i.e., colonoscopy). As expected, when we dropped our subsequent adherence rates for FOBT and FIT, FIT-mid became less effective, though it remained dominant compared with no screening. In contrast, colonoscopy became the most effective strategy when the subsequent adherence rates for FOBT and FIT were dropped from 63% to 40%, though it was associated with an unattractive incremental cost per QALY. It is clear that further information on long-term adherence rates for annual stool-based tests are needed.

Our study has limitations. As with most economic evaluations, our results are limited by available evidence. The natural history of adenomas and their progression to cancer is not clearly known. However, we populated our model with the best available evidence including a systematic review of adenoma and CRC prevalence rates [31] and modeled new adenoma growth and adenoma progression over time to closely match high quality clinical datasets [9]–[11]. We did not model cancers arising from lesions other than adenomas. However, most CRCs arising in average risk individuals are believed to develop via the traditional adenoma-carcinoma sequence. A small proportion of CRC may develop from undetectable lesions (i.e., flat or depressed adenomas), and it is known that some interval cancers can arise through a rapid adenoma-carcinoma sequence between screening studies [67]. It should be noted that this potential issue would impact the effectiveness of all CRC screening modalities, and thus would be unlikely to impact the differential effectiveness between our modeled strategies. Given data limitations, we modeled identical CRC stage distributions for cancers detected using all of the stool-based strategies despite differences in testing characteristics. Given FIT's superior sensitivity compared to FOBT, patients diagnosed with CRC might be expected to have more earlier stage cancers, which again would make FIT appear more attractive. We assumed that the results of each screening test were independent of the prior test result. While not informed by evidence, it is possible that this is not entirely true; however, it is important to note that the results of our analysis were robust to small changes in the sensitivity and specificity of each of the screening tests. Finally, although we did model the most widely available and promising screening strategies, additional technologies are being developed and it is possible that other screening paradigms, including nurse-based endoscopy, may become viable in the future as a means to reduce the cost of delivering flexible sigmoidoscopy and potentially colonoscopy.

In conclusion, annual screening with FIT having test performance characteristics within the mid-range reported in the literature is both more effective and less costly than other CRC screening modalities, including FOBT and colonoscopy, and not screening for CRC. Even if this level of test performance is not attainable in clinical practice, annual screening with a lower performing FIT is still highly attractive with a cost per QALY gained of <CAN$5,000 compared to no screening. Our results are robust suggesting that screening for CRC with FIT should be considered the modality of choice for average risk patients between the ages of 50 and 75 in North America.

## Abbreviations

CRC: colorectal cancer
CTC: computed tomographic colonography
FDNA: fecal DNA assay
FOBT: fecal occult blood test
FIT: fecal immunochemical test
QALY: quality-adjusted life-year
RCT: randomized controlled trial
