# Correction: Colorectal Cancer Screening for Average-Risk North Americans: An Economic Evaluation

**DOI:** 10.1371/annotation/0fd49c83-2c6d-42b5-a8c1-45a0aaedaa77

**Published:** 2012-11-02

**Authors:** Steven J. Heitman, Robert J. Hilsden, Flora Au, Scot Dowden, Braden J. Manns

There are two numerical errors in the penultimate sentence of the abstract. The abstract should read "Among the lifetimes of 100,000 average-risk patients, the number of cancers could be reduced from 4,857 to 1,393 and the number of CRC deaths from 1,782 to 457, while saving CAN$68 per person." The error occurred as the numbers 1,393 and 1,782 were accidentally switched within the sentence. The correct numbers are outlined in Table 5 and properly described in the results and discussion sections. 

